# Prognostic relevance and performance characteristics of serum IGFBP‐2 and PAPP‐A in women with breast cancer: a long‐term Danish cohort study

**DOI:** 10.1002/cam4.1504

**Published:** 2018-05-03

**Authors:** Ulrick Espelund, Andrew G. Renehan, Søren Cold, Claus Oxvig, Lee Lancashire, Zhenqiang Su, Allan Flyvbjerg, Jan Frystyk

**Affiliations:** ^1^ Medical Research Laboratory Department of Clinical Medicine, Health Aarhus University Aarhus Denmark; ^2^ The Christie NHS Foundation Trust Division of Molecular and Clinical Cancer Sciences School of Medical Sciences Faculty of Biology, Medicine and Health University of Manchester Manchester Academic Health Science Centre Manchester UK; ^3^ Department of Oncology Odense University Hospital Odense Denmark; ^4^ Department of Molecular Biology and Genetics, Science and Technology Aarhus University Aarhus Denmark; ^5^ Clarivate Analytics London UK; ^6^ Steno Diabetes Center Copenhagen (SDCC) The Capital Region of Denmark and University of Copenhagen Copenhagen Denmark; ^7^ Department of Endocrinology Odense University Hospital Odense Denmark; ^8^ Department of Clinical Research Faculty of Health Sciences University of Southern Denmark Odense Denmark

**Keywords:** Breast neoplasms, insulin‐like growth factor‐binding protein 2, pregnancy‐associated plasma protein A

## Abstract

Measurement of circulating insulin‐like growth factors (IGFs), in particular IGF‐binding protein (IGFBP)‐2, at the time of diagnosis, is independently prognostic in many cancers, but its clinical performance against other routinely determined prognosticators has not been examined. We measured IGF‐I, IGF‐II, pro‐IGF‐II, IGF bioactivity, IGFBP‐2, ‐3, and pregnancy‐associated plasma protein A (PAPP‐A), an IGFBP regulator, in baseline samples of 301 women with breast cancer treated on four protocols (Odense, Denmark: 1993–1998). We evaluated performance characteristics (expressed as area under the curve, AUC) using Cox regression models to derive hazard ratios (HR) with 95% confidence intervals (CIs) for 10‐year recurrence‐free survival (RFS) and overall survival (OS), and compared those against the clinically used Nottingham Prognostic Index (NPI). We measured the same biomarkers in 531 noncancer individuals to assess multidimensional relationships (MDR), and evaluated additional prognostic models using survival artificial neural network (SANN) and survival support vector machines (SSVM), as these enhance capture of MDRs. For RFS, increasing concentrations of circulating IGFBP‐2 and PAPP‐A were independently prognostic [HR_biomarker doubling_: 1.474 (95% CIs: 1.160, 1.875, *P* = 0.002) and 1.952 (95% CIs: 1.364, 2.792, *P* < 0.001), respectively]. The AUC_RFS_ for NPI was 0.626 (Cox model), improving to 0.694 (*P* = 0.012) with the addition of IGFBP‐2 plus PAPP‐A. Derived AUC_RFS_ using SANN and SSVM did not perform superiorly. Similar patterns were observed for OS. These findings illustrate an important principle in biomarker qualification—measured circulating biomarkers may demonstrate independent prognostication, but this does not necessarily translate into substantial improvement in clinical performance.

## Introduction

The insulin‐like growth factors, IGF‐I and IGF‐II, circulate in high concentrations, but at the cellular level, only a small fraction is able to stimulate the IGF‐I receptor (IGF‐IR), the primary target of the IGFs. This is due to the presence of six high‐affinity binding proteins (IGFBPs), which are present in molar excess of the IGFs and furthermore bind the IGFs with an affinity that exceeds that of the IGF‐IR 1.

To activate the IGF‐IR, the IGFs need to dissociate from the IGFBPs. This process is enhanced by IGFBP proteases, which cleave the IGFBPs and thereby reduce their ligand affinity markedly. PAPP‐A is a metalloproteinase that cleaves a subset of IGFBPs, and thus functions as a growth‐promoting enzyme, releasing bioactive IGF in close proximity to the IGF‐IR 2. PAPP‐A is overexpressed in several tumor types, including breast cancer 3, but the prognostic significance of circulating PAPP‐A remains uncertain.

There are established multidimensional relationships between the members of the IGF system. For example, in serum, free IGF‐I is positively correlated with its total circulating concentration; IGF‐I and IGF‐II are positively correlated with IGFBP‐3, which is the major IGF‐carrier 1; mean levels of IGF‐I and IGF‐II decrease with age, whereas mean IGFBP‐2 levels increase with age 4; and IGF‐I has a nonlinear inverted “U” shaped relationship with BMI 5, 6.

Insulin‐like growth factor ligands have well‐established tumor developing properties at a cellular level 7 and in the circulation, the concentration of IGF‐I, and to a lesser extent that of IGFBP‐3, are associated with subsequent risk of developing prostate, pre‐ and postmenopausal breast cancer and colorectal cancer 8. However, the role of circulating IGF‐related peptides in patients after cancer diagnosis—for example as prognosticators—is unclear. Previous studies report that serum IGFBP‐2 concentrations are elevated in a stage‐dependent manner in patients with numerous malignancies, including colorectal 9, prostate 10, ovarian 11, and lung 12 cancers, and might be prognostic, while serum IGFBP‐3 is implicated as prognostic in patients with metastatic colorectal cancer undergoing chemotherapy 13.

The present authors previously compared a cohort of patients with early‐stage breast cancer treated at the Odense University Hospital, Denmark, with matched controls 14, and showed that serum levels of free IGF‐I and free IGF‐II were elevated, whereas the respective total IGF levels were lower in patients with cancer than in controls. The findings from this small‐scale, cross‐sectional study (43 cancers; 38 controls) suggested that consideration of multidimensional relationship (MDR) of the IGF system might yield additional insight compared with single components alone, as free IGF levels depend on total IGF and IGFBP concentrations as well as IGFBP‐protease activity 1, 2.

We have previously capitalized on the modeling of multidimensional relationships of the IGF system using machine‐driven approaches, such as artificial neural networks (ANN) 15, and demonstrated considerable improvements (over and above conventional regression models) in performance characteristics (and thus, potential clinical utility) after measuring multiple IGF‐related biomarkers in the detection of colorectal cancer.

Here, we extended the earlier report from the breast cancer cohort treated at Odense University Hospital; measured a panel of seven IGF‐related circulating biomarkers; performed prognostic modeling against long‐term survival; and then evaluated performance characteristics against the clinically used prognostic model, namely the Nottingham Prognostic Index (NPI). In addition to Cox regression models, we evaluated prognostic models using survival ANNs (SANNs) and survival support vector machines (SSVMs), as these enhance capture of multidimensional relationships.

## Methods

### Study design and patients

We recruited women undergoing primary breast cancer surgery or operative biopsy at Odense University Hospital in Denmark (1993–1998). Inclusion criteria were Danish citizenship and a postal address within the County of Funen for at least 1 year. Women with any previous cancer diagnosis were excluded. A fasting blood sample was drawn on the morning of surgery whenever possible (*n*, 154). For practical reasons, some patients contributed with a blood sample at least 1 month postoperatively (median 3 months) instead of preoperatively (*n*, 186). A few patients contributed with a blood sample on both occasions (*n*, 29). For each cancer patient, two healthy women of the same age and from the same geographical region were invited to serve as control subjects (*n*, 614). This matching was performed with the aid of the Danish Central Office of Civil Registration. A flow diagram deriving the cases and controls is shown in Fig. S1.

All breast cancer patients and control subjects participated in a research assessment visit, which included physical examination, anthropometric measures, and questionnaire. The latter was developed specifically for this cohort and included smoking habits, menopausal status, medication, medical history and body composition in the past. After surgery, tumors were described by size, histological type and grade 16, estrogen receptor status 17 and lymph node involvement. Each participant gave informed consent prior to inclusion, and the study was approved by the local ethics committee. The study was performed in accordance with the 1975 declaration of Helsinki.

### Outcome measures

Patients were followed for up to 10 years with regular clinical visits. Disease‐free survival was recorded for each individual until the end of the clinical follow‐up schedule. All‐cause mortality for patients with breast cancer was recorded until December 2010. All data was obtained from The Danish Breast Cancer Cooperative Group (DBCG), a Danish nationwide initiative comprising historical patient records on breast cancer disease, treatment, and outcome. All patients in this study were treated according to the DBCG 89 protocol (Fig. S2), a national guideline used to allocate patients with breast cancer to treatment from 1990 to 2000 18. In brief, patients were divided into four distinct treatment groups according to menopausal status, tumor size, malignancy grade, steroid receptor status of the tumor and lymph node involvement. These were Protocol A, no adjuvant therapy; Protocol B, ovarian radiation or cyclophosphamide–methotrexat–fluorouracil (CMF) chemotherapy every third week for a total of nine times; Protocol C, tamoxifen with or without megestrol for varying durations; and Protocol D, CMF or cyclophosphamide–epirubicin–fluorouracil (CEF) chemotherapy every third week for a total of nine times, with or without pamidronate (Fig. S3). Patients were allocated to treatment independently of study participation.

### Nottingham Prognostic Index

The Nottingham Prognostic Index (NPI) has been widely used by breast cancer clinicians across Europe since its early descriptions by Blamey in 1979 19 and subsequent modifications. Importantly, for the purpose of the present analysis, it provides 10‐year survival estimates contemporaneous with the treatment period of this study. NPI is calculated as: lymph node (LN) stage (1–3) + Grade (1–3) + maximum diameter (cm × 0.2), giving an observed range of NPI from 2.08 (LN negative, grade 1, 0.4 cm) to 6.8 (LN Stage 3, grade 3, size 4.9 cm). There are six NPI groups recognized by scores as: an excellent prognostic group (EPG) with an observed NPI range of 2.00 to ≤2.40, good (GPG) 2.41 to ≤3.40; moderate I (MPG I) 3.41 to ≤4.40, moderate II (MPG II) 4.41 to ≤5.40, poor (PPG) 5.41 to ≤6.40, and very poor (VPG) 6.41 to 8.00. For this analysis, the PPG and VPG were combined because of small sample sizes. The corresponding 10‐year survivals are 96%, 93%, 81%, 74%, 50%, and 38% 20.

### Serum assays

All methods are thoroughly described elsewhere 21. In brief, we measured IGF bioactivity by our in‐house KIRA assay, which is designed to measure the ability of a given sample to phosphorylate the IGF‐IR in cultured cells in vitro under physiological conditions. The assay was performed as originally described with modifications, please see 21. As the IGF‐IR can be activated by IGF‐I, IGF‐II, and pro‐IGF‐II, we nominated the output of the bioassay as bioactive IGF.

We used gel chromatography (FPLC) at low pH to separate IGF‐I, IGF‐II, and pro‐IGF‐II from the IGFBPs. This method is cumbersome, but nevertheless regarded as the gold standard for separating IGFs from IGFBPs 21. After FPLC, IGF‐I, IGF‐II, and pro‐IGF‐II were determined by time‐resolved immunofluorometric assays (TR‐IFMAs) developed and validated in our laboratory 21. IGFBP‐2 was measured by a TR‐IFMA as previously described 21.

IGFBP‐3 was measured by a commercial kit (# IS‐4430) from IDS (Immunodiagnostic Systems Nordic A/S, Copenhagen, Denmark), using the automated iSYS platform 22. The IGFBP‐3 kit was generously supplied by the manufacturer. PAPP‐A was measured by a commercial kit from Ansh Labs (Webster, TX), generously provided by the manufacturer. Routine biochemical measurements were performed at the hospital's laboratory using widely available automated assays. All samples were analyzed in a blinded fashion in random order.

### Model developments

We developed conventional Cox prediction models to estimate associations and their 95% confidence intervals (CIs) for age and NPI (as a dummy variable) and IGF‐related analytes (as continuous variables), and the survival endpoint of interest. Bootstrapping was used (1000 iterations) to estimate 95% confidence intervals (CIs).

In addition to the conventional survival models, we investigated (nonlinear) solutions that do not rely on assumptions such as proportional hazards. Such approaches have shown comparable results with classical methods for clinical data, and improved performances for high‐dimensional data, such as microarray 23. To this end, we developed SANN and SSVM models. The SANN model utilized the commonly used feed‐forward network structure consisting of three layers (input, hidden, and output). Here, the log hazard was modeled as a function of covariates with a single linear output. The SSVM model utilized a Gaussian kernel function with cost and gamma parameters set at 0.1 (varying these parameters did not cause significant differences in the results).

Associations between the SANN and SSVM based scores and the real patient time to event was evaluated by the C‐index (a measure of concordance between a predictive biomarker and censored survival outcome) 24, area under the curve (AUC) and hazard Ratio (HR) as calculated from Kaplan–Meier (KM) survival curves. The C‐index assesses the concordance between the predicted and observed survival, with a value of 0.5 indicating random predictions, and 1 for perfect predicted survival. KM curves were generated based on the model scores where patients were stratified into high and low risk according to the median prediction.

Model performance is often reported on the data used to fit the model, resulting in highly biased model estimates. To avoid this common pitfall, cross‐validated estimates of survival distributions for predicted risk groups were computed as described by Simon 25. Briefly, leave‐one‐out (LOO) cross‐validation was used, where in each iteration one sample was left out of model fitting and a survival risk model was developed. Risk groups were defined based on the median risk score in this training set. The model was then applied to the left out sample resulting in a risk score and assignment to a risk group. This process was repeated for each of the training loops so that each sample was left out once and therefore had been classified as high or low risk using a model that they were not part of in any way. Model estimates were then computed by grouping all left out samples together and are therefore cross‐validated and unbiased.

As IGF‐related peptides were the biomarkers of interest, we first set a core model of non‐IGF variables, namely age and NPI. We then added each of the following variables separately to the model: IGFBP‐2, PAPP‐A, BMI, IGF‐I, IGF‐II, pro‐IGF‐II, and IGFBP‐3.

### Statistical analyses

Baseline characteristics were compared by Kruskal–Wallis and chi‐squared tests as appropriate.

For all models, receiver operating characteristic (ROC) curves were generated from postregression and post‐training estimations where 95% confidence intervals were calculated from the bootstrapping process. Optimal sensitivities and specificities were derived from the ROC curves. The primary summary performance indicator of “accuracy” was denoted by the AUC—values of 1.0 and 0.5 being perfect and random discrimination, respectively. In addition to the training set estimations, the cross‐validated estimates were also calculated as described in the methods.

Survival artificial neural networks and SSVM models were compared statistically using their respective C‐indices by estimating the variance of each estimator and the covariance of the two estimators under comparison. A *z*‐score test is then constructed to compare the two sets of predicted scores 26.

For the main performance model (postestimations of the Cox model), the discriminatory performance was assessed using two methods: (1) the Hanley and McNeil 27, a widely used method for comparing ROCs using between‐area correlations assuming binomial distributions and comparing paired data; and (2) the method by Pencina et al. 28, which derives a net reclassification improvement (NRI) output, an index of net change in events versus nonevents detected, which in turn focuses on medical decision making. We argue that, for most clinical settings, a NRI value of greater than 10%, over and above the conventional prognostic model, indicates a promising biomarker or set of biomarkers. The validity of the proportional hazards assumption was tested using Schoenfeld residuals; no deviations from proportionality were identified. SANN and SSVM modeling used R (version 3.1.2, R Foundation for Statistical Computing, Vienna, Austria); other statistical analyses were carried out in STATA (version 12.0, College Station, TX).

## Results

### Baseline characteristics

The baseline patient, tumor, and treatment characteristics according to tertiles of IGFBP‐2 and PAPP‐A are shown in Table 1 for the 301 women (with complete NPI and IGF data) with early‐breast cancer treated by surgery (with and without adjuvant therapies) at the Odense University Hospital, from 1993 to 1998. The median age was 55 years; median BMI was 24.9 kg/m^2^; 71% were postmenopausal; tumors were ER and PR positive in 76% and 38%, respectively; 37% were node positive; median NPI was 3.3; approximately a half were surgically treated by mastectomy; and approximately a half had no adjuvant therapy.

**Table 1:** Baseline patient, tumor and treatment characteristics according to tertiles of IGFBP‐2 and PAPP‐A, Odense University Hospital Breast Cancer series, 1993–1998

	Totals	Tertile 1	Tertile 2	Tertile 3	*P*_trends_
IGFBP‐2					
IGFBP‐2 *μ*g/L	253 (170–358)	138 (114–167)	252 (224–281)	415 (358–488)	
*N*	301	99	100	102	
Median age (IQR) years	55 (50–62)	56 (50–63)	54 (50–61)	55 (48–63)	0.732^a^
Median BMI (IQR) kg/m^2^	24.9 (22.6–28.9)	27.9 (24.7–32.0)	24.7 (22.9–26.9)	22.8 (20.8–25.4)	0.0001^a^
Menopausal status					
Premenopausal	88 (29)	25 (25)	28 (28)	35 (34)	0.349^b^
Postmenopausal	213 (71)	74 (75)	72 (72)	67 (66)	
Tumor grade					
Grade 1	110 (36)	35 (35)	37 (37)	38 (37)	0.945^b^
Grade 2	144 (48)	49 (50)	49 (49)	46 (45)	
Grade 3	47 (16)	15 (15)	14 (14)	18 (18)	
Tumor ER status					
Positive	201 (76)	72 (84)	66 (74)	63 (71)	0.116^b^
Negative	63 (24)	14 (16)	23 (26)	26 (29)	
Unknown	37	13	11	13	
Tumor PR status					
Positive	98 (38)	28 (33)	35 (41)	35 (39)	0.575^b^
Negative	161 (62)	56 (67)	51 (59)	54 (61)	
Unknown	42	15	14	13	
Node status					
Positive	111 (37)	36 (36)	39 (39)	36 (35)	0.854^b^
Negative	190 (63)	63 (64)	61 (61)	66 (65)	
Median NPI score (IQR)	3.3 (2.8–4.4)	3.3 (3.1–4.4)	3.3 (2.8–4.4)	3.4 (2.5–4.4)	0.980^a^
Surgical treatment					
Lumpectomy	155 (52)	54 (55)	57 (57)	44 (43)	0.109^b^
Mastectomy	146 (48)	45 (45)	43 (43)	58 (57)	
Treatment protocol					
No adjuvant therapy	160 (53)	50 (51)	53 (53)	57 (56)	0.277^b^
Ovarian ablation	35 (12)	7 (7)	14 (14)	14 (14)	
Tamoxifen	59 (20)	27 (27)	17 (17)	15 (15)	
Chemotherapy	47 (16)	15 (15)	16 (16)	16 (16)	
PAPP‐A					
PAPP‐A *μ*g/L	0.82 (0.68–1.00)	0.63 (0.56–0.68)	0.81 (0.77–0.88)	1.10 (1.00–1.3)	
*N*	301	99	100	102	
Median age (IQR) years	55 (50–62)	54 (50–60)	57 (51–62)	56 (48–64)	0.297^a^
Median BMI (IQR) kg/m^2^	24.9 (22.6–28.9)	25.4 (22.9–29.7)	25.1 (22.8–29.1)	24.2 (22.0–27.1)	0.191^a^
Menopausal status					
Premenopausal	88 (29)	31 (31)	20 (20)	37 (36)	0.034^b^
Postmenopausal	213 (71)	68 (69)	80 (80)	65 (64)	
Tumor grade					
Grade 1	110 (37)	38 (38)	34 (34)	38 (37)	0.875^b^
Grade 2	144 (48)	48 (48)	50 (50)	46 (45)	
Grade 3	47 (16)	13 (13)	16 (16)	18 (18)	
Tumor ER status					
Positive	201 (76)	74 (82)	63 (72)	64 (74)	0.226^b^
Negative	63 (24)	16 (18)	25 (28)	22 (26)	
Unknown	37	9	12	16	
Tumor PR status					
Positive	98 (38)	26 (30)	38 (44)	34 (40)	0.122^b^
Negative	161 (62)	62 (70)	48 (56)	51 (60)	
Unknown	42	11	14	17	
Node status					
Positive	111 (37)	38 (38)	34 (34)	39 (38)	0.766^b^
Negative	190 (63)	61 (62)	66 (66)	63 (62)	
Median NPI score (IQR)	3.3 (2.8–4.4)	3.3 (2.8–4.4)	3.3 (3.1–4.3)	3.4 (2.5–4.4)	0.701^a^
Surgical treatment					
Lumpectomy	155 (52)	46 (46)	57 (57)	52 (51)	0.328^b^
Mastectomy	146 (48)	53 (54)	43 (43)	50 (49)	
Treatment protocol					
No adjuvant therapy	160 (53)	54 (55)	56 (56)	50 (49)	0.632^b^
Ovarian ablation	35 (12)	10 (10)	9 (9)	16 (16)	
Tamoxifen	59 (20)	21 (21)	21 (21)	17 (17)	
Chemotherapy	47 (16)	14 (14)	14 (14)	19 (19)	

BMI, body mass index; IQR, interquartile range; NPI, Nottingham Prognostic Index.

Values in parentheses are percentages unless otherwise stated. Total sum of percentages may not equal 100%.

a Kruskal–Wallis test.

b Chi‐squared test for multiple comparisons.

Across the tertiles of circulating IGFBP‐2, there was an inverse association with BMI (*P* = 0.0001), but no significant associations with other factors. Across the tertiles of circulating PAPP‐A, there were no significant associations with other factors.

### IGFs: cancer versus controls

The distributions of circulating IGFs, bioactive IGF, IGFBP‐3, and PAPP‐A, according to tertiles of IGFBP‐2 and PAPP‐A, for women with breast cancer versus controls (516 with complete IGF data), are shown in Table 2. For some of the measurements (i.e., total IGF‐I, IGFBP‐3, and PAPP‐A), we observed statistically significant but hardly biologically relevant differences between median values for women with cancer compared with well‐matched controls, whereas the remaining measurements (i.e., IGFBP‐2, bioactive IGF, total IGF‐II, and pro‐IGF‐II) did not differ between the two groups.

**Table 2:** Distributions of circulating insulin‐like growth factors (IGF), IGF‐binding proteins, and PAPP‐A, according to tertiles of IGFBP‐2 and PAPP‐A, for women with breast cancer treated at Odense University Hospital, 1993–1998, and controls

	Controls (*n* = 516)	Cancer (*n* = 301)	a *P*_versus controls_	IGFBP‐2			a *P*_across tertiles_
				Tertile 1	Tertile 2	Tertile 3	
IGFBP‐2 *μ*g/L	255 (176–366)	253 (170–358)	0.681	138 (114–167)	252 (224–281)	415 (358–488)	
Total IGF‐I *μ*g/L	87 (65–110)	92 (70–113)	0.032	102 (71–129)	92 (74–112)	88 (68–104)	0.022
IGF bioactivity *μ*g/L	1.19 (0.97–1.62)	1.15 (0.84–1.50)	0.141	1.34 (0.92–1.73)	1.25 (0.86–1.53)	0.98 (0.72–1.26)	0.009
Total IGF‐II *μ*g/L	572 (498–652)	585 (500–667)	0.276	621 (522–717)	596 (504–653)	540 (476–624)	0.0004
Pro‐IGF‐II *μ*g/L	142 (112–175)	146 (112–178)	0.528	150 (114–193)	153 (117–182)	137 (108–167)	0.023
IGFBP‐3 *μ*g/L	4000 (347–4505)	4113 (3578–4599)	0.044	4450 (4048–5009)	4173 (3585–4534)	3689 (3354–4231)	0.0001
PAPP‐A *μ*g/L	0.80 (0.64–0.96)	0.82 (0.68–1.00)	0.030	0.75 (0.64–0.91)	0.79 (0.67–0.95)	0.94 (0.74–1.14)	0.0001

				PAPP‐A			a *P*_across tertiles_
				Tertile 1	Tertile 2	Tertile 3	
PAPP‐A *μ*g/L				0.63 (0.56–0.68)	0.81 (0.77–0.88)	1.10 (1.00–1.3)	
Total IGF‐I *μ*g/L				98 (74–119)	88 (70–114)	93 (68–108)	0.185
IGF bioactivity *μ*g/L				1.15 (0.86–1.43)	1.20 (0.86–1.71)	1.10 (0.82–1.54)	0.794
Total IGF‐II *μ*g/L				611 (528–693)	594 (519–662)	536 (470–625)	0.0047
Pro‐IGF‐II *μ*g/L				153 (120–192)	150 (113–178)	131 (104–168)	0.0165
IGFBP‐2 *μ*g/L				231 (137–301)	226 (167–344)	323 (219–416)	0.0001
IGFBP‐3 *μ*g/L				4167 (3647–4799)	4173 (3605–4648)	3937 (3492–4430)	0.063

a Kruskal–Wallis test.

Across the tertiles of circulating IGFBP‐2, there was an inverse association with total IGF‐II (*P* = 0.0004); an inverse association with IGFBP‐3 (*P* = 0.0001), and a positive association with PAPP‐A (*P* = 0.0001). Across the tertiles of circulating PAPP‐A, there was an inverse association with total IGF‐II (*P* = 0.0047) and pro‐IGF‐II (*P* = 0.0165). The correlations between the IGF‐related peptides in women with breast cancer (Table S2) broadly mirrored those in women without cancer (Table S3).

### Cox models: recurrence‐free survival

With a median follow‐up of 68 months, there were 105 recurrent events (total RFS events were 120). The 5‐ and 10‐year RFS rates were 77 (95% CIs: 72–81) percent and 55 (95% CIs: 48–61) percent, respectively. We screened various patient and tumor‐related factors, and all seven IGF‐related peptides as potential prognosticators for RFS using Cox regression models. By univariate analyses (Table 3), the following were significant: age (*P* = 0.049); highest versus lowest NPI category (*P* = 0.006); IGFBP‐2 (*P* = 0.002); and PAPP‐A (*P* < 0.001). We included these significant variables in the multivariate analysis—all remained significant, though generally with attenuation of effect size. By multivariate models, we additionally tested treatment‐related factors—but these did not turn out significant (Table S4).

**Table 3:** Univariate and multivariate modeling for circulating IGF‐related peptides with recurrence‐free survival^a^ as endpoint, Odense University Hospital Breast Cancer series, 1993–1998

	Incremental unit	Univariate			Multivariate		
		Hazard ratio	95% CI	*P* value	Hazard ratio	95% CI	*P* value
Age	Per 10 years	1.258	1.001, 1.581	0.049	1.248	0.995, 1.567	0.055
BMI	Per 5 kg/m^2^	0.865	0.718, 1.042	0.129			
Menopausal status							
Premenopausal		1.000	Referent				
Postmenopausal		1.281	0.839, 1.955	0.252			
Nottingham Prognostic Index							
Category 1	2.00–2.40^b^	1.000	Referent		1.000	Referent	
Category 2	2.41–3.40^b^	0.670	0.409, 1.097	0.112	0.768	0.466, 1.265	0.300
Category 3	3.41–4.40^b^	0.789	0.446, 1.395	0.416	0.956	0.534, 1.711	0.880
Category 4	4.41–5.40^b^	1.008	0.569, 1.784	0.977	1.163	0.651, 2.077	0.611
Category 5	5.41–8.00^b^	2.383	1.288, 4.408	0.006	2.385	1.278, 4.451	0.006
IGF‐related peptides^c^							
Total IGF‐I *μ*g/L	Per doubling	0.902	0.664, 1.227	0.515			
IGF bioactivity *μ*g/L	Per doubling	0.754	0.494, 1.150	0.190			
Total IGF‐II *μ*g/L	Per doubling	1.054	0.598, 1.860	0.855			
Pro‐IGF‐II *μ*g/L	Per doubling	0.801	0.582, 1.102	0.173			
IGFBP‐2 *μ*g/L	Per doubling	1.474	1.160, 1.875	0.002	1.397	1.089, 1.793	0.008
IGFBP‐3 *μ*g/L	Per doubling	0.903	0.493, 1.654	0.742			
PAPP‐A *μ*g/L	Per doubling	1.952	1.364, 2.792	<0.001	1.595	1.114, 2.282	0.011

BMI, body mass index; CI, confidence intervals; NPI, Nottingham Prognostic Index.

All analyses were performed as Cox regression models.

a Events for recurrence‐free survival were any recurrent disease or death, whichever came first.

b NPI scores range from 2.00 to 8.00.

c All IGF‐related peptide distributions log‐transformed to base 2.

**Figure 1:**
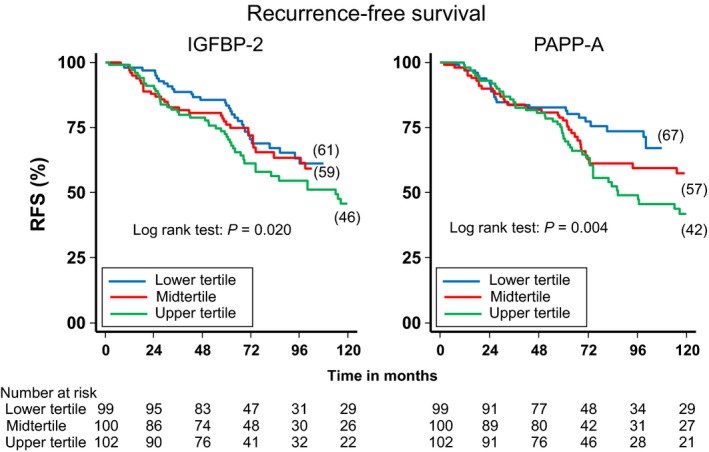
The recurrence‐free survival according to tertiles of IGFBP‐2 (left graph) and PAPP‐A (right graph). Blue: upper tertile, red: midtertile, and green: upper tertile.

To illustrate the potential clinical utility of IGFBP‐2 and PAPP‐A as prognostic biomarkers, we constructed Kaplan–Meier curves for RFS according to tertiles of IGFBP‐2 and PAPP‐A (Fig. 1). For IGFBP‐2, the discrimination for 10‐year RFS was modest—61%, 59%, and 46%, for the low, mid, and high tertile, respectively (*P* = 0.020). For PAPP‐A, the discrimination for 10‐year RFS was moderately better—67%, 57%, and 42%, for the low, mid, and high tertile, respectively (*P* = 0.004).

**Figure 2:**
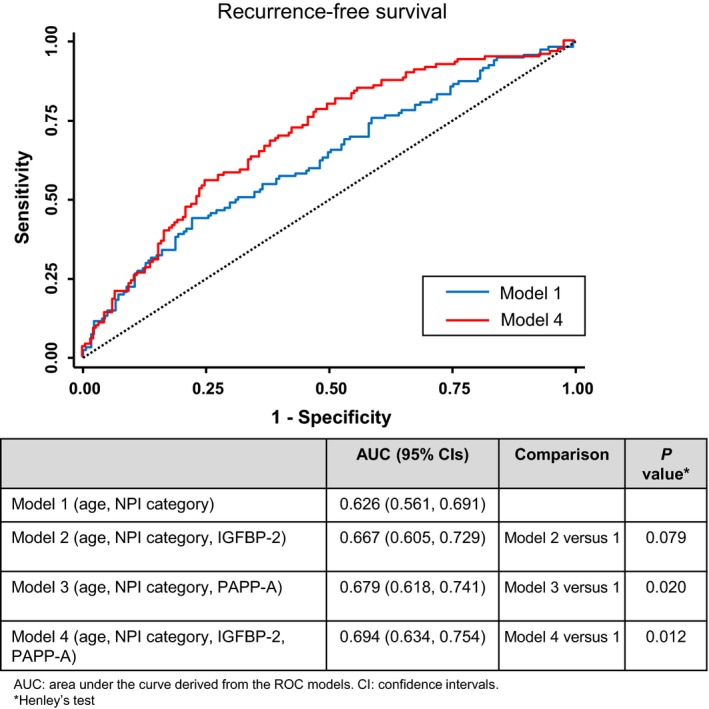
Receiver operating characteristic (ROC) curves of model 1 (blue) and model 4 (red). The models and their AUCs are shown in the Table below the graph.

Second, to predict for RFS, we explored the performance characteristics of models including IGFBP‐2 and PAPP‐A (Fig. 2). The core model (model 1) was exclusively based on clinically available parameters—age and NPI. For model 1, the AUC was 0.626. We initially added IGFBP‐2 and PAPP‐A, separately, with modest improvements in AUC compared with model 1 (model 2 vs. 1, *P* = 0.079; model 3 vs. 1, *P* = 0.020). We then added IGFBP‐2 plus PAPP‐A to the core model, yielding an AUC of 0.694 (model 4 vs. 1, *P* = 0.012).

We tested additional models including BMI (as BMI was inversely associated with IGFBP‐2), but there was no material improvement in performance. We also tested models with six IGF‐related peptides (excluding IGF bioactivity because this measurement was only determined in a subgroup of patients) and again, and there was no material improvement in performance (Table S5).

We then explored an additional decision tool, namely the NRI in a model that included age, NPI, IGFBP‐2, and PAPP‐A (the latter two log‐transformed). We explored various combinations of cutoffs for the two IGF‐related biomarkers at 40%, 50%, and 60% of the respective distributions. In the main, NRI values were equal to or less than the a priori 10% threshold, with one exception for cutoffs 40% and 50% for IGFBP‐2 and PAPP‐A, respectively, where the NRI was 14% (*P* = 0.042), however, with a wide standard error (Table S6). Accordingly, this result might have occurred by chance due to multiple testing.

### Machine‐Learning‐driven models: recurrence‐free survival

With RFS as the endpoint, we repeated the above performance characteristics analyses using the SANN and SSVM models. We speculated that these machine‐driven approaches might better capture the MDR of the IGF‐related peptides 15. The optimal models for SANN and SSVM, in the testing sets, yielded AUCs of 0.665 and 0.690 for RFS as endpoint (Table 4)—in other words, there was no material improvement in performance over the Cox model.

**Table 4:** Performance characteristics for models derived by Cox models, survival artificial neural networks (SANN), and survival support vector machines (SSVM), for recurrence‐free survival and overall survival

	Training set	Testing set
	AUC (95% CIs)	AUC (95% CIs)
Recurrence‐free survival		
Cox model		
Model 1 (age, NPI category)	0.626 (0.561–0.691)	
Model 4 (age, NPI category, IGFBP‐2, PAPP‐A)	0.694 (0.634–0.754)	
SANN		
Model 1 (age, NPI category)	0.699 (0.697–0.702)	0.648 (0.645–0.652)
Model 4 (age, NPI category, IGFBP‐2, PAPP‐A)	0.757 (0.754–0.759)	0.665 (0.661–0.669)
SSVM		
Model 1 (age, NPI category)	0.611 (0.608–0.614)	0.606 (0.603–0.609)
Model 4 (age, NPI category, IGFBP‐2, PAPP‐A)	0.690 (0.688–0.693)	0.690 (0.687–0.693)
Overall survival		
Cox model		
Model 1 (age, NPI category)	0.607 (0.543–0.670)	
Model 4 (age, NPI category, IGFBP‐2, PAPP‐A)	0.677 (0.616–0.738)	
SANN		
Model 1 (age, NPI category)	0.715 (0.712–0.718)	0.652 (0.648–0.656)
Model 4 (age, NPI category, IGFBP‐2, PAPP‐A)	0.780 (0.778–0.782)	0.670 (0.666–0.674)
SSVM		
Model 1 (age, NPI category)	0.631 (0.628–0.634)	0.634 (0.631–0.368)
Model 4 (age, NPI category, IGFBP‐2, PAPP‐A)	0.721 (0.719–0.724)	0.725 (0.722–0.728)

IGFBP‐2, insulin‐like growth factor‐binding protein 2; NPI, Nottingham Prognostic Index; PAPP‐A, pregnancy‐associated plasma protein A.

### Overall survival

From the 301 women, there were 107 deaths during follow‐up. The 5‐ and 10‐year OS rates were 90 (95% CIs: 87–93) percent and 79 (95% CIs: 74–83) percent, respectively. We repeated the same analyses as was performed for RFS. Results were similar to those for RFS. After screening, again similar variables were significant or borderline significant by univariate analysis—namely age (*P* = 0.053); highest versus lowest NPI category (*P* < 0.001); IGFBP‐2 (*P* = 0.017); and PAPP‐A (*P* = 0.001), and negatively with pro‐IGF‐II (*P* = 0.025). In the multivariate analysis, effects of these factors were generally attenuated, with significance remaining for age and highest NPI category (Table S7). As above, we explored the performance characteristics of models, including IGFBP‐2 and PAPP‐A, to predict for OS. The AUC for the core model (model 1: age and NPI) was 0.607, increasingly modestly to 0.677 with the addition of IGFBP‐2 and PAPP‐A, combined (Table 4). SANN and SSVM models added no additional performance enhancement for OS.

### Additional analyses

Recurrence after initial treatment for breast cancer may occur after a long disease‐free period—a biological process known as dormancy 29. We tested whether or not circulating IGF‐peptides differentially impacted on recurrent disease events in the first five years versus those manifesting after 5 years, by repeating our recurrence‐free analysis truncated at 60 months. The previously observed associations were broadly unchanged, with the exception that PAPP‐A was not significant in this model, whereas circulating IGFBP‐2 remained an independently prognostic factor (Table S8).

We explored the influence of menopausal status in all our models; it was not a significant prognosticator (data not shown).

## Discussion

### Main findings

Among women with long‐term follow‐up after breast cancer treatment, increasing concentrations of circulating IGFBP‐2 and PAPP‐A, determined at start of treatment, were independently associated with adverse prognosis; but when these biomarkers were tested against a clinically used prognostic model, namely NPI, there was only a modest improvement in performance characteristics. We speculated that machine‐driven approaches might enhance performance characteristics, by better capturing multidimensional relationships, but here too, improvements beyond routinely used prognostic models were only modest. These findings illustrate an important principle in biomarker qualification—measured circulating proteins, such as IGF‐related proteins, may demonstrate independent prognostication, but this does not necessarily translate into substantial improvement in clinical performance.

### Limitations and strengths

There are a number of potential limitations. First, the sample size was relatively small—we countered this by selecting RFS as the primary endpoint, which accumulated a large number of events over the long follow‐up. Second, there was lack of external validation of the model on an independent dataset. To partly address this, we used an approach of running 100 iterations on randomly selected proportions of the data and demonstrated consistency of our results. Third, machine‐learning methods identify data patterns by knowledge acquisition through an iterative learning process that allows development of nonlinear models and may intrinsically be disadvantaged by overfitting and high variability in the imputed data. In this analysis, overfitting was minimized with appropriate regularisation, early stopping, and robust cross‐validation 30.

There are several strengths. First, we used a dataset of IGF‐related peptides, which were validated in previous publications and consistent with the literature in terms of inter‐relationships between IGFs and with age and BMI 9, 31, 32, 33. Second, we used the gold method (acid chromatography) to separate IGFs from the IGFBPs and assayed serum IGF‐I and IGF‐II against WHO reference preparations as assay calibrators. This is likely to reduce the assay dependence of our results—a phenomenon that is well‐recognized for in particular IGF‐I measurements 34, 35. Third, we derived values for accuracy, sensitivity, and specificity, necessary for clinical decision making. Finally, we also derived an alternate decision tool—net reclassification improvement—and showed this to be modest.

### Findings in the context of other studies

In relation to serum IGFBP‐2, the findings in this study confirm observations in other reports that baseline IGFBP‐2 in patients with cancer is prognostic for poorer outcome in patients with cervical 36, pancreatic 37, and ovarian 11 cancer. Previous studies show that circulating IGFBP‐2 levels in women with ovarian cancer correlate with tumour tissue IGFBP‐2 mRNA levels 38, and in patients with colorectal cancer undergoing resection, elevated presurgery concentrations return to normal after resection 9. Furthermore, in ascites from women with ovarian cancer, IGFBP‐2 levels were higher and correlated positively with serum IGFBP‐2 39. Collectively, these studies suggest that circulating IGFBP‐2 levels partly reflect tumor secreted protein.

The findings for IGFBP‐2 and PAPP‐A contrast with the remainder of studied IGF variables; that is, IGF‐I, IGF‐II, pro‐IGF‐II, bioactive IGF, and IGFBP‐3. The latter biomarkers have no impact on survival in univariate analyses. This might seem contradictory to a large volume of epidemiology linking circulating total IGF‐I with increased risk of pre‐ and postmenopausal breast cancer, and circulating IGFBP‐3 and postmenopausal breast cancer 8. However, in the postdiagnosis setting, several studies have investigated the relationship between circulating IGFs and breast cancer survival, with inconsistent findings. In 110 postmenopausal women following breast cancer surgery, Pasanisi et al. 40 reported higher IGF‐I levels among women who developed recurrences compared with recurrence‐free (mean follow‐up: 5.5 years), but these differences were not statistically significant. In 600 women from the HEAL trial, where biomarkers were measured at 30 months postoperatively, Duggan et al. 41 reported significant associations between IGF‐I, IGF‐I to IGFBP‐3 ratio (as an approximation of free IGF‐I), and all‐cause mortality. However, the number of events was small (42 deaths). We found no association between total IGF‐I and survival, and no association when we directly determined IGF bioactivity (albeit in a subpopulation of our total cohort). Similarly, Goodwin et al. 42 measured circulating IGF‐I, IGF‐II, and IGFBP‐3 at 4–12 weeks postoperatively in 512 women with early‐stage breast cancer and found no association with survival.

### Biological plausibility

In the present study, the Cox regression analyses identified serum PAPP‐A and IGFBP‐2 as independent prognostications of both RFS and OS. However, inclusion of IGFBP‐2 and PAPP‐A only marginally increased the predictive value above that of the NPI. Furthermore, the baseline concentrations of IGFBP‐2 and PAPP‐A were virtually similar when comparing patients with cancer and healthy controls. Thus, one could argue that the NPI is already embedding the prognostic impact of IGFBP‐2 and PAPP‐A, explaining why neither PAPP‐A nor IGFBP‐2 had any major impact as prognosticators when added to the NPI. Obviously, the present study does not allow us to draw such conclusions, but we do believe it is possible to justify the idea that PAPP‐A and IGFBP‐2 are involved in breast cancer. Many studies support that IGFBP‐2 expression plays an oncogenic role in breast cancer 43. IGFBP‐2 is consistently expressed in most breast cancer tissues and expression levels associate with the grade of malignancy 43. Even though there may be no overall relation between IGFBP‐2 staining intensity and survival, the survival among patients with ER‐negative/IGFBP‐2‐positive breast cancer tissue was impaired 44. Furthermore, we have previously shown that IGFBP‐2 in breast cancer cells was a marker of resistance to anti‐estrogen therapy, whereas cell proliferation did not depend on IGFBP‐2 expression 45.

The association between IGFBP‐2 and breast cancer appears to be intimately linked to the tumor suppressor phosphatase and tensin homolog (PTEN), which interacts with IGFBP‐2 in an IGF‐independent manner 43, 46. During normal conditions, PTEN limits the activation of the PI3K/AKT/mTOR pathway, thereby serving as an anabolic brake on cell proliferation and survival [46. During malignant transformation, inactivating mutations of PTEN are frequent events. The functional lack of PTEN may lead to unopposed proliferation and anti‐apoptosis via the PI3K/AKT/mTOR pathway. Interestingly, when not occupied by IGF‐II, IGFBP‐2 has been shown to suppress PTEN in breast cancer cells 47. Thus, the association between an elevated serum (free) IGFBP‐2 concentration and a poorer prognosis is likely to reflect an increased tumor synthesis of IGFBP‐2, a suppressed PTEN level, and consequently an unopposed tumor growth.

PAPP‐A was the only other IGF‐related protein that associated with RFS: an elevated level predicting a poorer prognosis. PAPP‐A is an IGFBP‐specific protease, having IGFBP‐2 as a substrate. Therefore, the current observation of a positive correlation between serum concentrations of IGFBP‐2 and PAPP‐A may appear counterintuitive. However, one has to remember that the ability of PAPP‐A to cleave IGFBP‐2 is IGF‐dependent, and even following binding of IGF, PAPP‐A cleavage of IGFBP‐2 is a relatively slow process compared to cleavage of its other substrates 48. Indeed, we have recent data indicating that PAPP‐A only affects IGFBP‐2 marginally. In pleural effusions, where PAPP‐A is close to 50‐fold elevated as compared to serum, the degradation of IGFBP‐2 remains similar to that in serum 49 and in serum from lung cancer patients the major part of IGFBP‐2 circulates in its free form (data in preparation). On the basis of our recent and present data, we speculate that in vivo, IGFBP‐2 is primarily unoccupied and hence not only protected against PAPP‐A degradation but also able to suppress PTEN 46, 47. Another issue that remains to be investigated is whether the relationship between PAPP‐A and IGFBP‐2 serum concentrations is causal.

We noted that serum PAPP‐A was independently prognostic for RFS in the long‐term (full) model but not in the 5‐year right‐truncated model, suggesting that PAPP‐A might have less influence in early recurrent disease events, although sample sizes were too small to make definitive conclusions. However, as we found no previous reports evaluating the association between prognosis and serum PAPP‐A in patients with breast cancer, we have no data for comparison. Nevertheless, our results are not surprising given the laboratory data—that (1) PAPP‐A is frequently overexpressed in tumors, including breast cancer 3; and (2) in vivo, PAPP‐A overexpressing SKOV3 clones (ovarian cancer) have accelerated tumor growth compared with mutant PAPP‐A and controls 50. Investigation of angiogenesis indicates that overexpression of PAPP‐A favors development of mature tumor vasculature 50. The potential role of PAPP‐A in breast cancer recurrence is further supported, albeit indirectly, by studies of stanniocalcin‐2, which recently was discovered to inhibit the proteolytic activity of PAPP‐A, and hence its ability to liberate IGF‐I 51. Thus, in a study of primary breast cancer tumors, Joensuu et al. found a higher expression level of stanniocalcin‐2 in tumors which showed a very late relapse. As the relationship between PAPP‐A and stanniocalcin‐2 was not known at that time point, the authors suggested that stanniocalcin‐2 acted as a survival factor for breast cancer cells 52. However, knowing that stanniocalcin‐2 inhibits the enzymatic function of PAPP‐A, one may argue that by obstructing PAPP‐A, stanniocalcin‐2 was indeed delaying the recurrence of breast cancer rather than serving as a survival factor. Thus, even though PAPP‐A in the present study was unable to serve as an additional prognosticator of breast cancer recurrence, we believe it may still play a pathogenic role.

### Implications and future studies

The parallel development of biomarker(s) and novel anticancer therapies involves several stages—including discovery, qualification, and validation—as outlined, for example by the Cancer Research UK biomarker roadmap (http://www.cancerresearchuk.org/prod_consump/groups/cr_common/@fre/@fun/documents/generalcontent/cr_027486.pdf).As part of qualification, the sensitivity, specificity, and accuracy of a biomarker assay are compared with established thresholds for minimally acceptable clinical performance criteria. It is likely that single circulating biomarkers may not be sufficiently informative alone, whereas multiple biomarkers in combination with additional types of biomarkers (e.g., imaging modalities) may yield clinically relevant information. This study shows that despite initial promise from the prognostic modeling, serum IGFBP‐2 and PAPP‐A fell short of steep changes in clinical performance. If serum IGFBP‐2 and PAPP‐A were to be pursued as clinical biomarkers, alternative studies might be to look at repeated measures and whether or not changes in values were prognostic. Equally, it is important to make decisions when to proceed no further with development of some biomarkers.

## Conflict of Interest

The authors declare that there is no conflict of interest that would prejudice the impartiality of this scientific work.

## Supporting information


**Figure S1.** Flow diagram to case control analysis.
**Figure S2.** DBCG 89 protocol.
**Figure S3.** Treatment types (A to D).
**Table S1.** Characteristics of 549 women with breast cancer with and without blood samples.
**Table S2.** Spearman correlations between IGF biomarkers among 301 women with breast cancer.
**Table S3.** Spearman correlations between IGF biomarkers among 516 women without cancer.
**Table S4.** Multivariate modelling patient, NPI stage and treatment types with recurrence‐free survival* as endpoint, Odense University Hospital Breast Cancer series, 1993–1998.
**Table S5.** Performance characteristics for models derived by Cox models including all seven IGF‐related peptides for recurrence‐free survival as endpoint (*n* = 301).
**Table S6.** Pancina's method to derive Net Reclassification Improvement (NRI) with recurrence‐free survival as endpoint.
**Table S7.** Univariate and multivariate modelling for circulating IGF‐related peptides with overall survival* as endpoint, Odense University Hospital Breast Cancer series, 1993–1998.
**Table S8.** Univariate and multivariate modelling for circulating IGF‐related peptides with recurrence‐free survival* as endpoint, right truncated at 60 months, Odense University Hospital Breast Cancer series, 1993–1998.
