# Supplementary material for: Prognostic relevance and performance characteristics of serum IGFBP‐2 and PAPP‐A in women with breast cancer: a long‐term Danish cohort study
Source: Cancer Med. 2018 May 3;7(6):2391–404. doi: 10.1002/cam4.1504 (PMC6010701; doi:10.1002/cam4.1504)
Supplement: Supplementary file 1 — Figure S1. Flow diagram to case control analysis. Figure S2. DBCG 89 protocol. Figure S3. Treatment types (A to D). Table S1. Characteristics of 549 women with breast cancer with and without blood samples. Table S2. Spearman correlations between IGF biomarkers among 301 women with breast cancer. Table S3. Spearman correlations between IGF biomarkers among 516 women without cancer. Table S4. Multivariate modelling patient, NPI stage and treatment types with recurrence‐free survival* as endpoint, Odense University Hospital Breast Cancer series, 1993–1998. Table S5. Performance characteristics for models derived by Cox models including all seven IGF‐related peptides for recurrence‐free survival as endpoint (n = 301). Table S6. Pancina's method to derive Net Reclassification Improvement (NRI) with recurrence‐free survival as endpoint. Table S7. Univariate and multivariate modelling for circulating IGF‐related peptides with overall survival* as endpoint, Odense University Hospital Breast Cancer series, 1993–1998. Table S8. Univariate and multivariate modelling for circulating IGF‐related peptides with recurrence‐free survival* as endpoint, right truncated at 60 months, Odense University Hospital Breast Cancer series, 1993–1998. [file CAM4-7-2391-s001.docx]

**Electronic supplementary material**

**Prognostic relevance and performance characteristics of serum IGFBP-2 and PAPP-A in women with breast cancer: a long-term Danish cohort study**

**Ulrick Espelund,^1^ Andrew G Renehan,^2^ Søren Cold,^3^ Claus Oxvig,^4^ Lee Lancashire,^5^ Zhenqiang Su,^5^ Allan Flyvbjerg,^1,6^ Jan Frystyk ^1,7^**

^1^ Medical Research Laboratory, Department of Clinical Medicine, Health, Aarhus University, Denmark

^2^ Division of Molecular & Clinical Cancer Sciences, School of Medical Sciences, Faculty of Biology, Medicine and Health, University of Manchester, Manchester, United Kingdom

^3^ Department of Oncology, Odense University Hospital, Denmark

^4^ Department of Molecular Biology and Genetics, Science & Technology, Aarhus University, Denmark

^5^ Clarivate Analytics, London, United Kingdom

^6^ Steno Diabetes Center Copenhagen (SDCC), The Capital Region of Denmark and University of Copenhagen, Copenhagen, Denmark

^7^ Department of Endocrinology and Internal Medicine, Aarhus University Hospital, Denmark.

**Corresponding author:**

Professor Jan Frystyk

The Medical Research Laboratory

Department of Clinical Medicine, Aarhus University

Aarhus University Hospital
Nørrebrogade 44

DK-8000 Aarhus C

Denmark

Cell phone: +45 2330 0330

e-mail: [frystyk@clin.au.dk](mailto:frystyk@clin.au.dk)

Initial invited

1509

**Figure**

**S1**

**Flow diagram to**

**case**

**-**

**control**

**analysis.**

DBCG:

Danish Breast Cancer Cooperative Group

IGF: insulin

-

like growth factors. NPI: Nottingham Prognostic Index.

Initial eligibility

449

Declined, 255

No contact, 7

Benign pathology,

629

Non

-

citizenship

, 13

Non

-

Odense

address, 6

Other cancer, 15

Non

-

DBCG

, 112

Miscellaneous, 23

Total cases

340

No sample, 109

Complete NPI & IGF

for analysis

301

I

ncomplete IGF NPI

data

, 39

Initial invited

1460

Initial eligibility

771

Declined,

561

No contact,

84

Non

-

citizenship

, 9

Non

-

Odense

address, 2

Other cancer, 25

Miscellaneous, 8

Total controls

614

No sample, 157

Complete IGFs for

analysis

516

Incomplete

IGF

data

, 98

**Cases**

**Controls**

**Table S1 Characteristics of 549 women with breast cancer with and without blood samples**

|  |  | **Eligible with sample** | **Eligible without sample** |
| --- | --- | --- | --- |
|  |  |  |  |
| No. of patients |  | 340 | 109 |
| Median age (IQR) | years | 55 (50-62) | 55 (50-63) |
| Pre-menopausal | n (%) | 94 (28) | 31 (28) |
| Lumpectomy | n (%) | 174 (51) | 61 (56) |
| Treatment protocol  (A/ B/ C/ D) | n (%) | 189/ 37/ 65/ 49  (56/ 11/ 19/ 14) | 65/ 9/ 18/ 17  (60/ 8/ 17/ 16) |
| Median tumour size (IQR) | mm | 15 (11 – 22) | 14 (9-20) |
| Tumour grade  (I/ II/ III/ unknown) | n (%) | 97/ 124/ 47/ 72  (29/ 36/ 14/ 21) | 26/ 41/ 18/ 24  (24/ 38/ 17/ 22) |
| Steroid receptor positivity | n (%) | 269 (79) | 84 (77) |
| Lymph node positivity | n (%) | 120 (35) | 34 (31) |
| Screen detected | n (%) | 187 (55) | 57 (52) |
| Surgery on Odense | n (%) | 336 (99) | 102 (94) |
| Oncology treatment in Odense | n (%) | 339 (100) | 107 (98) |
|  |  |  |  |

IQR: interquartile range

**Study design and patients**

We derived a cohort with a complete set of IGF analytes and tumour characteristics to patient-level Nottingham Prognostic Index (NPI), based within the Danish Breast Cancer Cooperative Group study. From an initial 1509 women, we recruited those undergoing primary breast cancer surgery or operative biopsy at Odense University Hospital, Denmark (1993 to 1998), with Danish citizenship and a postal address within the County of Funen for at least one year. Women with a previous cancer diagnosis or two neoplastic pathologies were excluded. From these, a fasting blood sample was drawn on the morning of surgery. Of the initial 449 eligible women, samples were available or adequate in 340 women (Figure S1). There were no material differences in patient, tumour and treatment characteristics for the 340 versus the 109 women who had no samples (Table S1).

Among the 340 women with samples, blood was collected whenever possible pre-operatively, in 154 women (referred to as “PreOP”), and for practical reasons, 186 women contributed their blood sample post-operatively (median: 3 months: referred to as “PostOP”). Twenty-nine women contributed samples as PreOP and PostOP. There was strong concordance between pre- and post-operative levels of all IGF analytes, except for total IGF-II (mean levels were higher in the PostOP group versus PreOP group, p < 0.001). From the 340 women, there were 301 women with a complete set of NPI scores, and IGF analytes and these form the basis of this analysis.

For each cancer patient, approximately two healthy women of the same age and from the same geographical region were invited as control subjects. This matching was performed with the aid of the Danish Central Office of Civil Registration. From the initial 1460 invitees, there were 614 controls with samples (Figure S1). Of these, there were 516 women with a complete set of IGF analytes.

All breast cancer patients and control subjects participated in a research assessment visit, which included physical examination, anthropometric measures, and questionnaire. The latter was developed specifically for this cohort and included smoking habits, menopausal status, medication, medical history, and body composition in the past. After surgery, tumours were described by size, histological type and grade, oestrogen receptor status and lymph node involvement. Each participant gave informed consent prior to inclusion and the study was approved by the local ethics committee. The study was performed in accordance with the 1975 declaration of Helsinki.

**
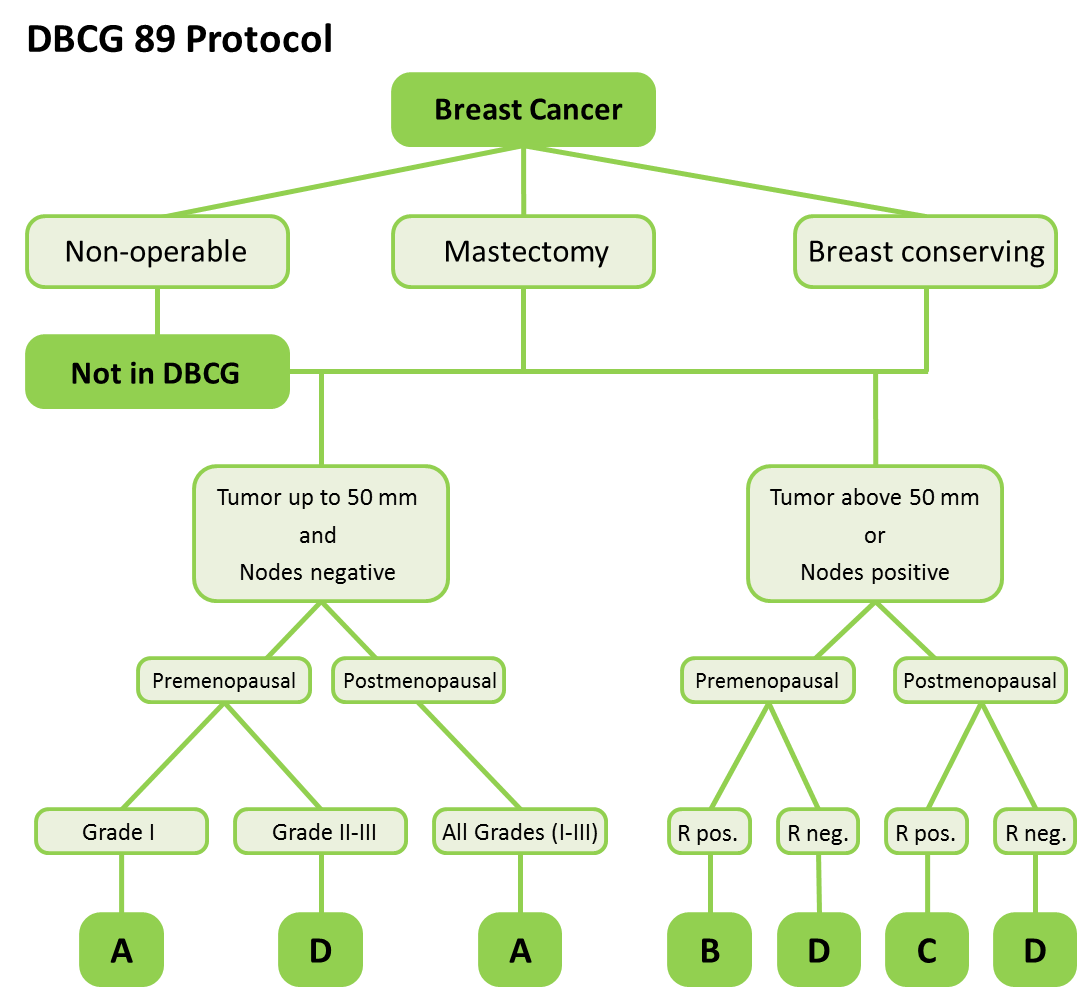
Figure S2 DBCG 89 Protocol**

**
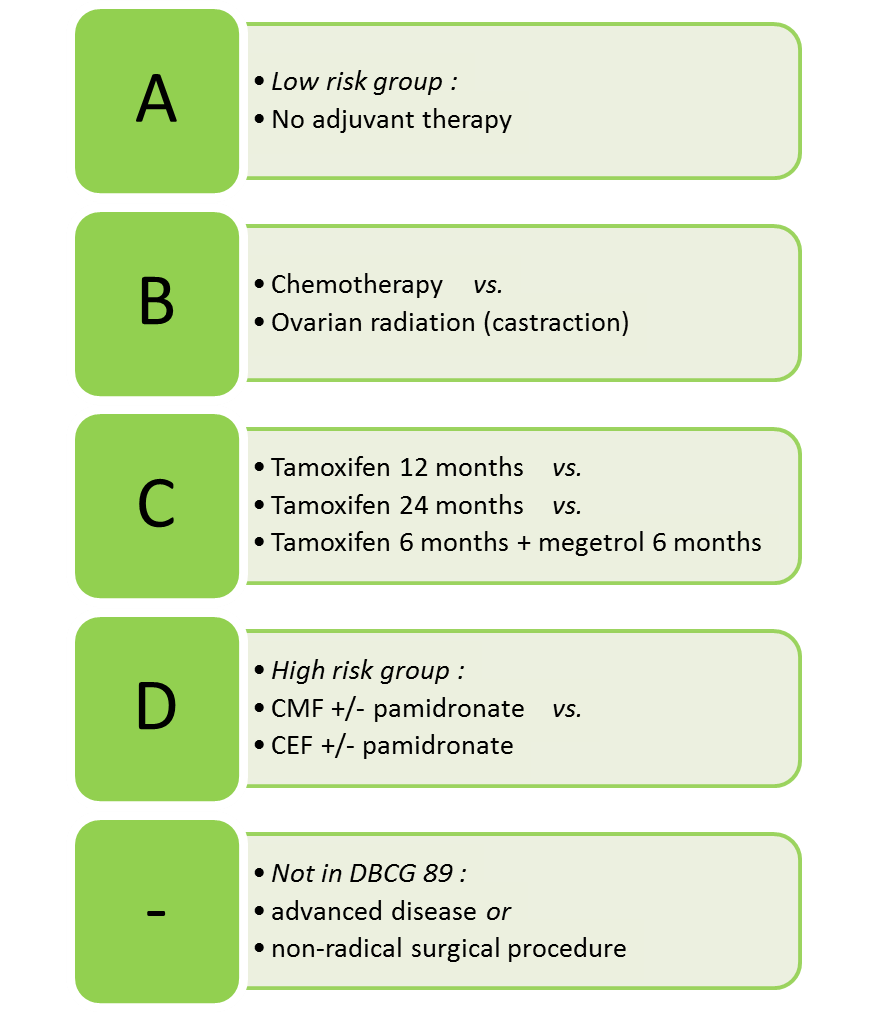
Figure S3 – Treatment types (A to D)**

**Table S2 Spearman correlations between IGF biomarkers among 301 women with breast cancer**

Data are Rho and P-values. *IGF-I bioactivity analysed separately based on 131 patients.

|  | **IGFBP-2** | **Total IGF-I** | **IGF bioactivity** | **Total IGF-II** | **Pro-IGF-II** | **IGFBP-3** | **PAPP-A** |
| --- | --- | --- | --- | --- | --- | --- | --- |
| **IGFBP-2** | 1.00 |  |  |  |  |  |  |
| **Total IGF-I** | -0.14  0.015 | 1.00 |  |  |  |  |  |
| **IGF bioactivity** | -0.23*  0.007 | 0.49*  <0.0001 | 1.00 |  |  |  |  |
| **Total IGF-II** | -0.24  <0.0001 | 0.39  <0.0001 | 0.05*  0.611 | 1.00 |  |  |  |
| **Pro-IGF-II** | -0.14  0.017 | 0.38  <0.0001 | 0.21*  0.019 | 0.54  <0.0001 | 1.00 |  |  |
| **IGFBP-3** | -0.40  <0.0001 | 0.51  <0.0001 | 0.25*  0.004 | 0.76  <0.0001 | 0.47  <0.0001 | 1.00 |  |
| **PAPP-A** | 0.26  <0.0001 | -0.07  0.245 | 0.05  0.572 | -0.17  0.005 | -0.16  0.005 | -0.09  0.112 | 1.00 |

**Table S3 Spearman correlations between IGF biomarkers among 516 women without cancer**

Data are Rho and P-values. *IGF-I bioactivity analysed separately based on 159 individuals.

|  | **IGFBP-2** | **Total IGF-I** | **IGF-bioactivity** | **Total IGF-II** | **Pro-IGF-II** | **IGFBP-3** | **PAPP-A** |
| --- | --- | --- | --- | --- | --- | --- | --- |
| **IGFBP-2** | 1.00 |  |  |  |  |  |  |
| **Total IGF-I** | -0.20  <0.0001 | 1.00 |  |  |  |  |  |
| **IGF bioactivity** | -0.23*  0.004 | 0.52*  <0.0001 | 1.00 |  |  |  |  |
| **Total IGF-II** | -0.17  0.0002 | 0.46  <0.0001 | 0.26*  0.001 | 1.00 |  |  |  |
| **Pro-IGF-II** | -0.17  0.0001 | 0.37  <0.0001 | 0.25*  0.001 | 0.56  <0.0001 | 1.00 |  |  |
| **IGFBP-3** | -0.27  <0.0001 | 0.43  <0.0001 | 0.38*  <0.0001 | 0.67  <0.0001 | 0.44  <0.0001 | 1.00 |  |
| **PAPP-A** | 0.25  <0.0001 | -0.03  0.438 | -0.01*  0.925 | -0.01  0.865 | -0.08  0.055 | -0.06  0.220 | 1.00 |

**Table S4 Multivariate modelling patient, NPI stage and treatment types with recurrence-free survival* as endpoint, Odense University Hospital Breast Cancer series, 1993-1998**

|  |  | **Multivariate** | | |
| --- | --- | --- | --- | --- |
|  | **Incremental unit** | Hazard  ratio | 95% CI | P value |
|  |  |  |  |  |
| **Age** | per 10 years | 1.336 | 0.992,1.799 | 0.056 |
| **Nottingham Prognostic Index** |  |  |  |  |
| Category 1 | 2.00 to 2.40† | 1.000 | referent |  |
| Category 2 | 2.41 to 3.40† | 0.703 | 0.418, 1.180 | 0.183 |
| Category 3 | 3.41 to 4.40† | 0.892 | 0.477, 1.668 | 0.721 |
| Category 4 | 4.41 to 5.40† | 1.127 | 0.581, 2.188 | 0.722 |
| Category 5 | 5.41 to 8.00† | 2.518 | 1.138, 5.571 | 0.023 |
|  |  |  |  |  |
| **Treatment types** |  |  |  |  |
| No adjuvant therapy |  | 1.000 | referent |  |
| Ovarian ablation |  | 0.962 | 0.422, 2.190 | 0.926 |
| Tamoxifen |  | 0.816 | 0.459, 1.451 | 0.488 |
| Chemotherapy |  | 1.299 | 0.637, 2.647 | 0.471 |
|  |  |  |  |  |

Analyses were performed as Cox regression models.

NPI: Nottingham Prognostic Index. CI: confidence intervals.

*Events for recurrence-free survival were any recurrent disease or death, whichever came first.

†NPI scores ranges from 2.00 to 8.00.

**Table S5 Performance characteristics for models derived by Cox models including all seven IGF-related peptides for recurrence-free survival as endpoint (n = 301)**

|  | AUC (95% CIs) |
| --- | --- |
| **Recurrence-free survival** |  |
| Model 1 (age, NPI category) | 0.626  (0.561-0.691) |
| Model 4 (age, NPI category, IGFBP-2, PAPP-A) | 0.694  (0.634-0.754) |
| Model 6 (age, NPI category, IGFBP-2, PAPP-A, total IGF-I, IGF bioactivity, total IGF-II, pro-IGF-II, IGFBP-3) | 0.696  (0.635-0.757) |
|  |  |

NPI: Nottingham Prognostic Index.

IGFBP-2: insulin-like growth factor binding protein 2. PAPP-A: pregnancy-associated plasma protein A

**Table S6 Pancina’s method to derive Net Reclassification Improvement (NRI) with recurrence-free survival as endpoint**

|  | **Distribution cut-offs** | |  |  |
| --- | --- | --- | --- | --- |
|  | **IGFBP-2** | **PAPP-A** | **NRI (se)** | **P value** |
| Round 1 | 40% | 60% | 10 (7) | 0.127 |
|  | 50% | 60% | 6 (6) | 0.345 |
|  | 60% | 60% | Not estimable |  |
| Round 2 | 40% | 60% | 10 (7) | 0.127 |
|  | 40% | 50% | 14 (7) | 0.042 |
|  | 40% | 40% | Not estimable |  |
| Round 3 | 60% | 60% | Not estimable |  |
|  | 60% | 50% | 6 (6) | 0.345 |
|  | 60% | 40% | 10 (7) | 0.127 |

**Table S7 Univariate and multivariate modelling for circulating IGF-related peptides with overall survival* as endpoint, Odense University Hospital Breast Cancer series, 1993-1998**

|  |  | **Univariate** | | |  | **Multivariate** | | |
| --- | --- | --- | --- | --- | --- | --- | --- | --- |
|  | **Incremental unit** | Hazard  ratio | 95% CI | P value |  | Hazard  ratio | 95% CI | P value |
|  |  |  |  |  |  |  |  |  |
| **Age** | per 10 years | 1.255 | 0.997, 1.581 | 0.053 |  | 1.373 | 1.059, 1.779 | 0.016 |
| **BMI** | per 5 kg/m^2^ | 0.929 | 0.758, 1.138 | 0.477 |  |  |  |  |
| **Menopausal status** |  |  |  |  |  |  |  |  |
| Pre-menopausal |  | 1.000 | referent |  |  |  |  |  |
| Post-menopausal |  | 1.342 | 0.852, 2.114 | 0.204 |  |  |  |  |
| **Nottingham Prognostic Index** |  |  |  |  |  |  |  |  |
| Category 1 | 2.00 to 2.40† | 1.000 | referent |  |  | 1.000 | referent |  |
| Category 2 | 2.41 to 3.40† | 1.081 | 0.602, 1.941 | 0.794 |  | 1.193 | 0.643, 2.212 | 0.574 |
| Category 3 | 3.41 to 4.40† | 0.983 | 0.501, 1.927 | 0.960 |  | 1.331 | 0.648, 2.735 | 0.435 |
| Category 4 | 4.41 to 5.40† | 1.839 | 0.965, 3.507 | 0.064 |  | 2.120 | 1.080, 4.160 | 0.029 |
| Category 5 | 5.41 to 8.00† | 3.261 | 1.679, 6.334 | <0.001 |  | 2.715 | 1.294, 5.694 | 0.008 |
|  |  |  |  |  |  |  |  |  |
| **IGF-related peptides‡** |  |  |  |  |  |  |  |  |
| Total IGF-I μg/l | per doubling | 0.730 | 0.521, 1.022 | 0.067 |  |  |  |  |
| IGF bioactivity μg/l | per doubling | 0.634 | 0.406, 0.989 | 0.045 |  |  |  |  |
| Total IGF-II μg/l | per doubling | 0.789 | 0.427, 1.462 | 0.453 |  |  |  |  |
| Pro-IGF-II μg/l | per doubling | 0.662 | 0.461, 0.949 | 0.025 |  | 0.867 | 0.587, 1.281 | 0.475 |
| IGFBP-2 μg/l | per doubling | 1.375 | 1.058, 1.788 | 0.017 |  | 1.311 | 0.979, 1.753 | 0.068 |
| IGFBP-3 μg/l | per doubling | 0.910 | 0.473, 1.749 | 0.778 |  |  |  |  |
| PAPP-A μg/l | per doubling | 1.849 | 1.276, 2.679 | 0.001 |  | 1.459 | 0.972, 2.191 | 0.068 |
|  |  |  |  |  |  |  |  |  |

All analyses were performed as Cox regression models.

NPI: Nottingham Prognostic Index. BMI: body mass index. CI: confidence intervals.

*Events for overall survival were death, any cause.

†NPI scores ranges from 2.00 to 8.00.

‡all IGF-related peptide distributions log transformed to base 2.

**Table S8 Univariate and multivariate modelling for circulating IGF-related peptides with recurrence-free survival* as endpoint, right truncated at 60 months, Odense University Hospital Breast Cancer series, 1993-1998**

|  |  | **Univariate** | | |  | **Multivariate** | | |
| --- | --- | --- | --- | --- | --- | --- | --- | --- |
|  | **Incremental unit** | Hazard  ratio | 95% CI | P value |  | Hazard  ratio | 95% CI | P value |
|  |  |  |  |  |  |  |  |  |
| **Age** | per 10 years | 1.018 | 0.762, 1.360 | 0.902 |  |  |  |  |
| **BMI** | per 5 kg/m^2^ | 0.886 | 0.692, 1.134 | 0.338 |  |  |  |  |
| **Menopausal status** |  |  |  |  |  |  |  |  |
| Pre-menopausal |  | 1.000 | referent |  |  |  |  |  |
| Post-menopausal |  | 0.911 | 0.539, 1.537 | 0.727 |  |  |  |  |
| **Nottingham Prognostic Index** |  |  |  |  |  |  |  |  |
| Category 1 | 2.00 to 2.40† | 1.000 | referent |  |  | 1.000 | referent |  |
| Category 2 | 2.41 to 3.40† | 0.541 | 0.254, 1.151 | 0.111 |  | 0.582 | 0.273, 1.241 | 0.161 |
| Category 3 | 3.41 to 4.40† | 1.087 | 0.517, 2.279 | 0.826 |  | 1.171 | 0.557, 2.462 | 0.677 |
| Category 4 | 4.41 to 5.40† | 1.664 | 0.812, 3.411 | 0.164 |  | 1.688 | 0.822, 3.468 | 0.154 |
| Category 5 | 5.41 to 8.00† | 2.379 | 1.055, 5.362 | 0.037 |  | 2.554 | 1.118, 5.835 | 0.026 |
|  |  |  |  |  |  |  |  |  |
| **IGF-related peptides‡** |  |  |  |  |  |  |  |  |
| Total IGF-I μg/l | per doubling | 0.934 | 0.611, 1.428 | 0.754 |  |  |  |  |
| IGF bioactivity μg/l | per doubling | 0.798 | 0.462, 1.377 | 0.417 |  |  |  |  |
| Total IGF-II μg/l | per doubling | 1.056 | 0.487, 2.291 | 0.890 |  |  |  |  |
| Pro-IGF-II μg/l | per doubling | 0.929 | 0.591, 1.461 | 0.751 |  |  |  |  |
| IGFBP-2 μg/l | per doubling | 1.513 | 1.101, 2.081 | 0.011 |  | 1.472 | 1.062, 2.042 | 0.020 |
| IGFBP-3 μg/l | per doubling | 0.948 | 0.422, 2.128 | 0.897 |  |  |  |  |
| PAPP-A μg/l | per doubling | 1.552 | 0.963, 2.501 | 0.071 |  | 1.169 | 0.720, 1.899 | 0.562 |
|  |  |  |  |  |  |  |  |  |

All analyses were performed as Cox regression models.

NPI: Nottingham Prognostic Index. BMI: body mass index. CI: confidence intervals.

*Events for recurrence-free survival were any recurrent disease or death, whichever came first.

†NPI scores ranges from 2.00 to 8.00.

‡all IGF-related peptide distributions log transformed to base 2.
